# Supplementary material for: Using Sodium Polyacrylate to Gel-Spin Lignin/Poly(Vinyl Alcohol) Fiber at High Lignin Content
Source: Polymers (Basel). 2022 Jul 4;14(13):2736. doi: 10.3390/polym14132736 (PMC9269501; doi:10.3390/polym14132736)
Supplement: Supplementary file 1 [file polymers-14-02736-s001.zip › polymers-1712609-supplementary.pdf]

Using Sodium Polyacrylate to Gel-Spin Lignin/Poly (Vinyl Alcohol) Fiber at High Lignin Content

Manik Chandra Biswas[a] and Ericka Ford[b]

[a] Department of Textile Engineering, Chemistry and Science, North Carolina State University, 1020 Main Campus Drive, Raleigh, NC, 27695, USA

[b] Department of Textile Engineering, Chemistry and Science, The Nonwovens Institute, North Carolina State University, 1020 Main Campus Drive, Raleigh, NC, 27695, USA

Supporting Information (SI)

Gel-spinning of Poly (Vinyl Alcohol) (PVA) Fiber

Gel-spinning involves several steps include polymer dissolution in appropriate solvent as first step. Polymer solution is extruded through a spinneret, an air gap and then a low temperature bath to form as-spun gel-fibers. The gel state helps to reduce chain entanglements to benefit better chain alignment. High ratios of fiber stretching are crucial to obtain highly crystalline and oriented fiber microstructures, which contributes to the superior mechanical performance of gel-spun fibers. Compared to melt-spun fibers, gel-spun fibers have higher draw-ability because of less chain entanglements and solvent facilitate chain extension in drawing.

A schematic of the gel-spinning process is shown in Fig. S1. Step 1 involves the loading of spinning dope (50 mL) into a high-pressure steel syringe, which was then heated to 85 °C with a constant voltage (120 V) heating belt and the extrusion of fiber through a 22-gauge (0.43 mm inner diameter) syringe needle. Neat PVA dope was spun at 3-5 mm air gap in methanol bath at negative temperature (-25 °C) (step 1). The resulting as-spun gel-fibers were collected onto a rotating winder and later immersed in the 5 °C coagulation bath for 24 h (step 2). Fibers were drawn through 1-4 stages of silicone oil at elevated temperatures (step 3).

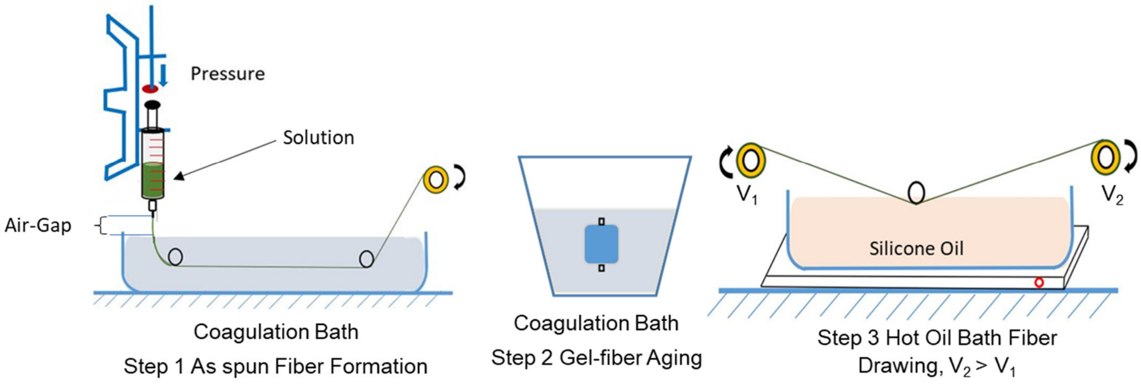

Figure S1. Gel spinning of fibers is shown as three steps: as-spun gel formation (Step 1), aging of gel-fibers (Step 2), and multistage thermal drawing of fibers (Step 3).

1.1. Process Conditions of PVA gel-spinning

Process parameters for lignin/PVA fibers that were drawn in methanol bath are summarized in Table SI1. As neat PVA fiber required overnight aging that avoids hot air

drawn steps. The as-spun gel fibers drawn in multiple stages after over-night aging in coagulation bath. At stage 1 drawing, fiber solidifies via diffusion of solvent to the high temperature oil bath.

Table S1. Drawing Parameters of Gel-Spun neat PVA

<table>
<tr><td colspan="2">Neat PVA</td><td>Single Filament</td></tr>
<tr><td colspan="2">As-Spun Draw Ratio (DR)
(3-5 mm Air Gap)</td><td>2.5</td></tr>
<tr><td rowspan="2">Stage 1 Drawing</td><td>Oil Temp. (^oC)</td><td>80</td></tr>
<tr><td>DR</td><td>4.2</td></tr>
<tr><td rowspan="2">Stage 2 Drawing</td><td>Oil Temp. (^oC)</td><td>100</td></tr>
<tr><td>DR</td><td>1.4</td></tr>
<tr><td rowspan="2">Stage 3 Drawing</td><td>Oil Temp. (^oC)</td><td>170</td></tr>
<tr><td>DR</td><td>1.3</td></tr>
<tr><td rowspan="2">Stage 4 Drawing</td><td>Oil Temp. (^oC)</td><td>210</td></tr>
<tr><td>DR</td><td>1.1</td></tr>
<tr><td colspan="2">*Total DR</td><td>21</td></tr>
<tr><td colspan="2">Effective Diameter(μm)</td><td>42</td></tr>
<tr><td colspan="2">Linear Density (dtex)</td><td>9.6</td></tr>
</table>

1.2. Mechanical Properties of Neat PVA Fibers

Mechanical properties of neat PVA fibers are tabulated in Table SI2. The neat PVA fibers exhibited tenacity 4.15 cN/dtex and tensile modulus 76 cN/dtex. Water is known to reduce the mechanical properties of PVA fibers, and so, the moisture resistivity of PVA

fibers was studied with fibers immersed in water and measured wet tensile strength (Table S2).

Table S2. Mechanical Properties of neat PVA and Additive Modified Fibers

Sample	Dry Condition				Wet Condition	
	**Specific Modulus (cN/dtex)**	**Tenacity (cN/dtex)**	**Strain at Break, (%)**	**Toughness J/g**	**Specific Modulus (cN/dtex)**	**Tenacity (cN/dtex)**
Neat PVA	76.00 ± 3.21	4.15 ± 0.08	8.78 ± 0.67	0.4 ± 0.03	14.00 ± 2.80	2.10 ± 0.07

The SEM images of neat PVA fiber fracture tips after mechanical testing shown in Fig. SI2

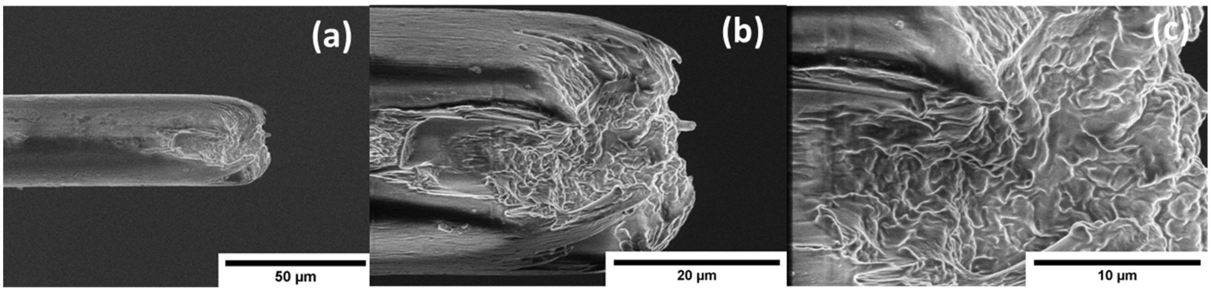

Figure S2. (a) Low to (b,c) high-resolution SEM images of fracture tips of neat PVA fiber from methanol coagulation bath

1.3. Effect of SPA and Lignin on PVA fiber structure

WAXD was used to study structural changes in fully drawn fiber. The most prominent diffraction peak of neat PVA fiber is shown in Fig. S3, is due to overlapping ($10\bar{1}$) and (101) peaks at $2\theta = 19.6°$.

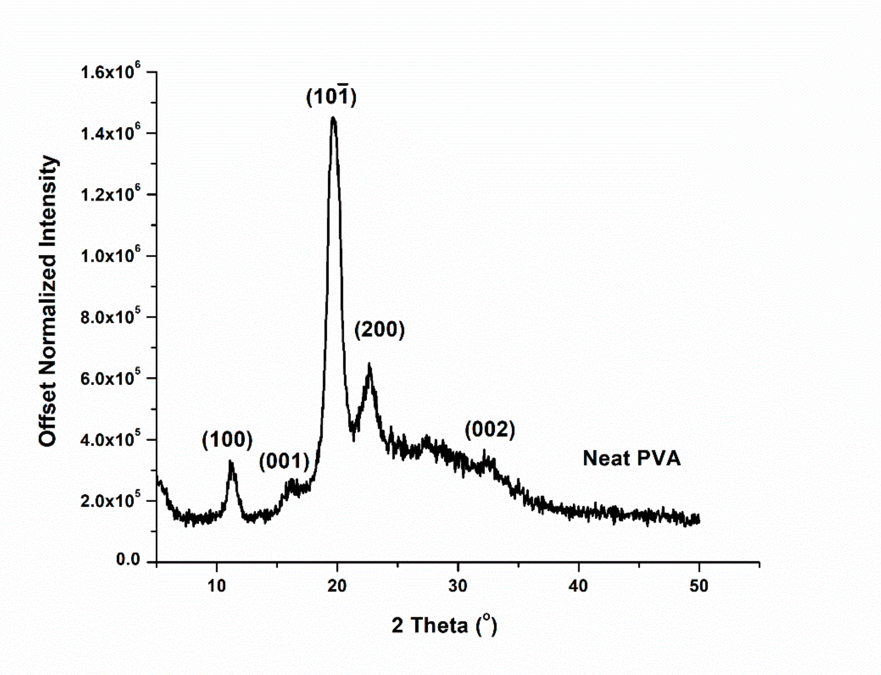

Figure S3. WAXD diffractograms of neat PVA gel fibers.

Figure S4 showed IR absorbance spectra of neat PVA gel-spun fiber where the absorbance peak of -OH peak shifted from 3304 cm^{-1} for neat PVA to 3284 cm^{-1} for 30% lignin induced fiber, implies highest intermolecular interaction. The characteristic crystalline peak of neat PVA observed at 1141 cm^{-1}.

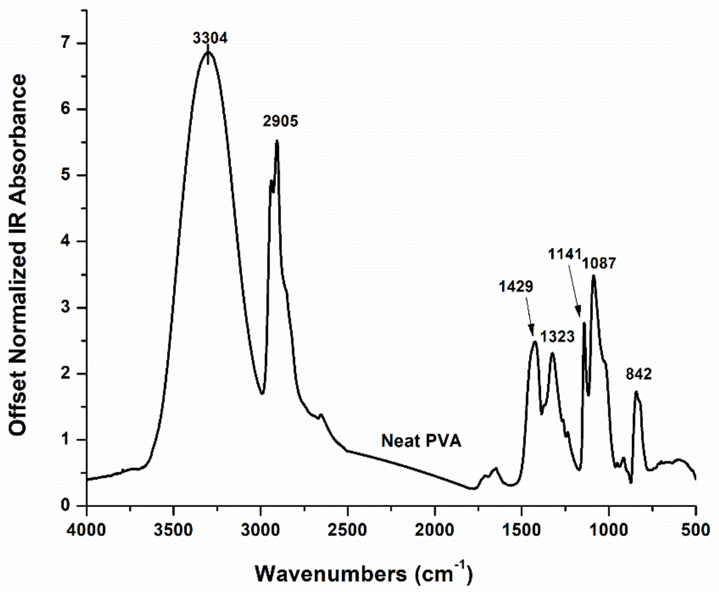

Figure S4. IR absorbance spectra of neat PVA fibers

1.4. Moisture Sensitivity

The water resistance behavior of modified PVA fibers at room and elevated temperatures was monitored using optical microscopy. All fibers maintained structural integrity after immersion in room temperature water (Fig. S5 and Fig. 10). Wherein, neat PVA fiber exhibited small shrinkage at 85 ^{o}C implies temperature dependent dissolution evidenced by rough surface due to the aggregates of dissolved fibers (Fig. S5, a_2).

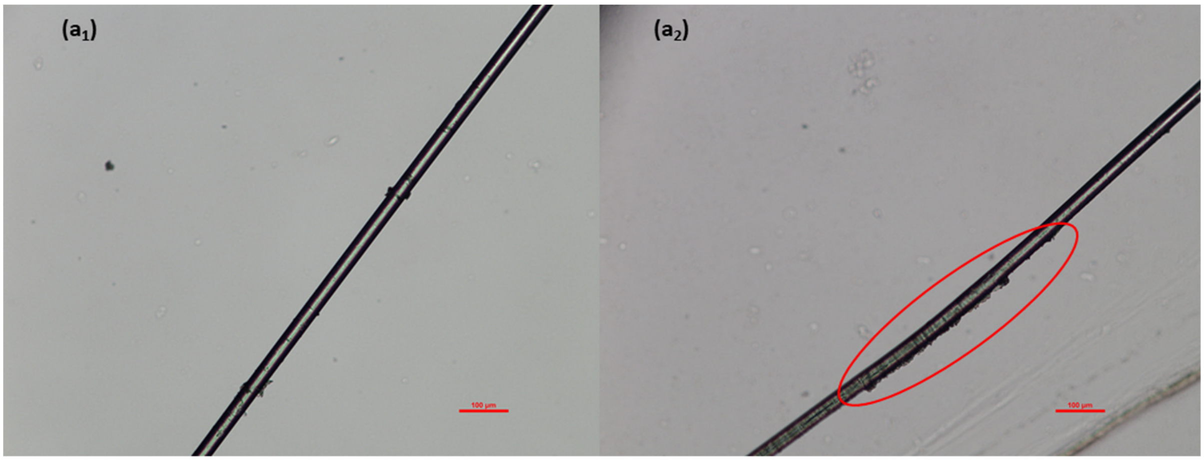

Figure S5. Optical Micrographs of (a) neat PVA fibers after immersion in water at (1) 25 ^{o}C and (2) 85 ^{o}C (100 μm scale bar)
